# Immune Effector Cell-Associated Neurotoxicity Syndrome After CAR T-Cell Therapy and Other Psychiatric Manifestations: A Review and Case Series

**DOI:** 10.3390/jcm14051451

**Published:** 2025-02-21

**Authors:** Adela Georgiana Buciuc, Sabrina Tran, Mary Weber, Vanessa Padilla, Maria Rueda-Lara, Zelde Espinel

**Affiliations:** 1 Department of Psychiatry and Behavioral Sciences, University of Miami Miller School of Medicine, Jackson Health System, Miami, FL 33136, USA; 2 Department of Psychiatry and Behavioral Sciences, University of Miami Miller School of Medicine, Miami, FL 33136, USA; sht36@med.miami.edu (S.T.); mxw808@miami.edu (M.W.); vpadilla@med.miami.edu (V.P.); mrueda2@med.miami.edu (M.R.-L.); z.espinel@miami.edu (Z.E.)

**Keywords:** psychiatric manifestations, CAR-T, immune effector cell-associated neurotoxicity syndrome, ICANS

## Abstract

**Background/Objectives**: Chimeric antigen receptor (CAR) T-cell therapy has transformed the treatment of hematologic malignancies, achieving durable remissions in cases refractory to standard therapies. A potentially life-threatening complication is immune effector cell-associated neurotoxicity syndrome (ICANS), which poses significant challenges to clinical management. ICANS encompasses a range of neuropsychiatric symptoms, including delirium, mood disorders, psychosis, seizures, and cerebral edema. The psychiatric dimensions of ICANS remain underreported, and their interplay with neurologic manifestations is poorly understood. This study reviews the psychiatric manifestations of ICANS and presents a case series illustrating its clinical complexity. **Methods**: A systematic literature search was conducted using PubMed and Google Scholar for studies published between 2020 and 2024. Search terms included “ICANS”, “delirium”, “CAR T-cell”, “neurotoxicity”, and “psychiatric”. The inclusion criteria included studies published in English that focused on adult patients experiencing neuropsychiatric symptoms of ICANS. Two clinical cases of ICANS with prominent psychiatric features are presented. **Results**: The literature review found three relevant studies, which emphasized agitation, hypoactivity, and mood disturbances as often-overlooked psychiatric symptoms linked to ICANS. The case series highlights psychiatric manifestations, including delirium, irritability, and cognitive impairment. Recovery was supported through interventions such as corticosteroid tapering, antipsychotic treatment, and multidisciplinary care. **Conclusions**: ICANS is a multifaceted syndrome with significant neuropsychiatric sequelae that complicate its diagnosis and management. An enhanced recognition of its psychiatric dimensions and interdisciplinary approaches are critical to improving outcomes.

## 1. Introduction

Chimeric antigen receptor (CAR) T-cell therapy is a transformative advancement in immunotherapy, in which autologous T-cells are genetically engineered to express chimeric antigen receptors targeting specific cancer antigens. This innovative approach has significantly improved clinical outcomes, particularly in individuals with refractory or relapsed hematologic malignancies, offering durable remissions when conventional therapies have failed. Despite its remarkable efficacy, CAR T-cell therapy is associated with a spectrum of immune-mediated toxicities. Neurotoxic adverse events related to CAR T-cell therapy are of concern and underexplored, posing significant challenges in clinical management.

One of the most concerning neurotoxic manifestations is immune effector cell-associated neurotoxicity syndrome (ICANS). This life-threatening immune-mediated toxicity can cause significant neuropsychiatric disturbances, including mood disorders, delirium, psychosis, seizures, cerebral edema, elevated intracranial pressure, and transient coma [1]. With an incidence of 26.9% for all-grade ICANS, it is crucial to gain a comprehensive understanding of the condition to improve patient outcomes [2]. The interplay between the neurologic and psychiatric manifestations of ICANS remains insufficiently understood, with psychiatric symptoms often underreported or misattributed to other factors. This knowledge gap complicates timely diagnosis and appropriate management, potentially exacerbating the morbidity risk. Addressing this issue calls for a multidisciplinary and integrative approach, blending expertise from immunology, neurology, and psychiatry to uncover the mechanisms behind ICANS and its varied presentations.

## 2. Materials and Methods

**Figure 1:**
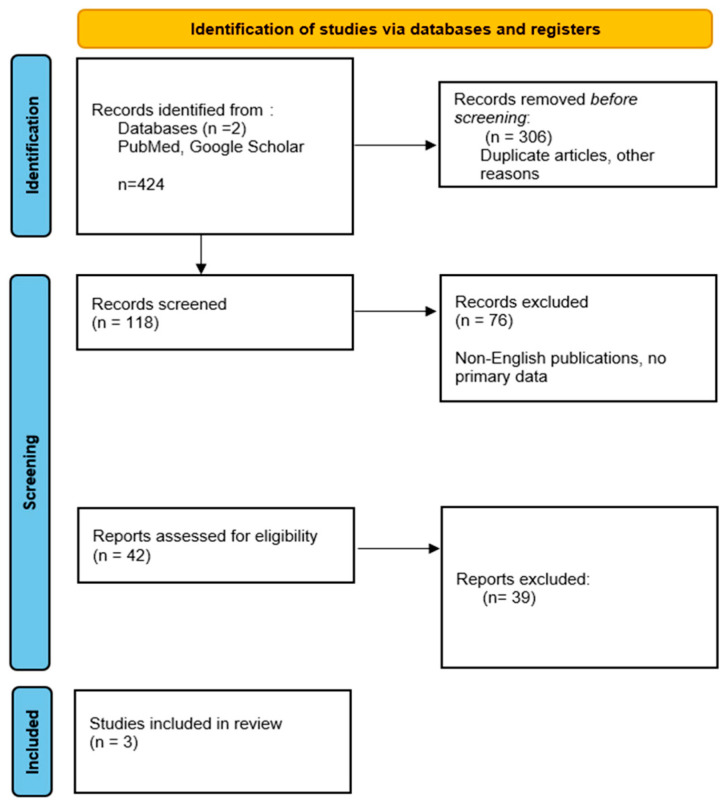
PRISMA flow chart [6].

A comprehensive literature search was conducted using PubMed and Google Scholar databases. Keywords included “ICANS”, “delirium”, “CAR T-cell”, “neurotoxicity”, and “psychiatric”. Inclusion criteria comprised published studies written in English from 2020 to 2024 that provided data on neuropsychiatric manifestations of ICANS. Publications in languages other than English, studies without primary data (i.e., reviews, editorials), and articles studying the pediatric population were excluded. A total of 424 articles were identified. After screening titles and abstracts, 42 were selected for full-text review. Finally, 3 studies met the inclusion criteria. Figure 1 presents more detailed information on the methods used. Table 1 showcases a summary of the included studies.

**Table 1:** An overview of the three studies included.

Serial Number	Authors and Year Published	Title	Number of Participants Included in the Studies (n)	Number of Patients with ICANS (n)	Neurocognitive Manifestations	**Other Psychiatric Manifestations Mentioned**
1	R. Mullane et al., 2020 [3]	Patient-Reported Neuropsychiatric Outcomes of Long-Term Survivors after Chimeric Antigen Receptor T Cell Therapy.	40	34	Difficulty concentrating and in word finding and memory	19 patients reported depression and/or anxiety
2	Maillet et al., 2021 [4]	Evaluation of mid-term (6–12 months) neurotoxicity in B-cell lymphoma patients treated with CAR T cells: a prospective cohort study.	24	12	Dyscalculia, dysgraphia, difficulties with executive function	Anxiety and/or depression
3	R. Quinn et al., 2021 [5]	Neurotoxicity of Axicabtagene Ciloleucel and Long-Term Outcomes—in a Minority Rich, Ethnically Diverse Real World Cohort	34	16	Disorientation, confusion, cognitive impairments in all cognitive domains	21 patients reported anxiety and/or depression

## 3. Results

### 3.1. Literature Review

The literature review revealed the high prevalence of ICANS among individuals receiving CAR T-cell therapy, with an overall rate of 81.63% across the studies analyzed. The most observed neurocognitive manifestations included deficits in attention, memory, and executive functioning, as well as more severe cognitive disruptions such as disorientation and confusion.

### 3.2. Case Presentations

**Case Study 1:**

A 68-year-old male with a history of mixed small and non-small cell lung cancer (NSCLC) with left frontal metastasis to brain status post resection, hypertension, hyperlipidemia, occasional alcohol use, and no prior psychiatric history was admitted for an evaluation of a two-week history of worsening confusion. His oncological treatment history included complications from chemotherapy and radiation therapy, resulting in radiation necrosis and immune effector cell-associated neurotoxicity syndrome (ICANS). Seizure prophylaxis with levetiracetam up to 1500 mg oral daily, alongside dexamethasone 40 mg oral daily, initially led to clinical improvement. A subsequent clinical decline prompted the patient’s family to taper levetiracetam while maintaining dexamethasone at a 20 mg daily dose for less than three months.

The patient exhibited marked agitation, prompting the primary oncology team to initiate olanzapine 15 mg oral daily and haloperidol 10 mg intravenously every 8 h as needed for behavioral disturbances. Psychiatry and oncology teams were consulted for further management.

During the psychiatric evaluation, the patient was oriented only to person. His ability to participate in a formal cognitive screening was limited due to pronounced irritability and inattention. As a non-native English speaker, he became easily frustrated when required to use a language translator. He exhibited deficits across multiple cognitive domains, including impaired attention, evidenced by an inability to direct, sustain, or shift focus during the evaluation. Memory impairments were noted, with difficulties in both immediate and delayed recall. His speech was slurred, and his language was impaired, characterized by limited fluency. He struggled to follow simple commands, and his thought process was tangential, making it difficult for him to maintain a coherent line of conversation. No delusions or hallucinations were elicited during the evaluation. A physical examination revealed bilateral upper extremity tremors and mild rigidity.

The laboratory findings were notable for elevated lactate levels (4.2 mmol/L) and thrombocytopenia. Electroencephalogram (EEG) demonstrated generalized cortical dysfunction, primarily affecting the left hemisphere, consistent with moderate encephalopathy. Magnetic resonance imaging (MRI) ruled out surgical indications for hydrocephalus, as determined by neurosurgery consultation. Neurology was consulted for concerns of encephalitis, and cerebrospinal fluid (CSF) analysis revealed no evidence of infection or malignancy.

The psychiatric assessment indicated a clinical presentation consistent with delirium, caused by multiple etiologies. Following psychiatric and neurologic interventions, including dexamethasone tapering to 4 mg daily and a reduction in olanzapine from 15 mg to 5 mg oral daily, the patient’s condition gradually improved. Delirium precautions, including early mobility, reducing polypharmacy, regulating sleep, and a multidisciplinary care approach, were crucial to his recovery.

**Case Study 2:**

A 73-year-old female with a history of uveal melanoma metastasized to the liver, hypertension, hyperlipidemia, and MALT lymphoma presented to the emergency department following a mechanical fall. The fall resulted in left tibial and fibular shaft fractures. She was found tachycardic, hypotensive, and with acute kidney injury due to urinary retention. No source of infection was identified. Her hospitalization involved orthopedic surgical intervention and the management of medical complications.

The patient had previously undergone immune checkpoint inhibitor (ICI) therapy for her metastatic melanoma but developed resistance to treatment, with progressive disease despite therapy. Due to the lack of alternative options and the failure of ICIs to control her malignancy, she was subsequently enrolled in CAR T-cell therapy. Following an earlier admission and autologous T-cell infusion, she developed late ICANS. Her stem cell treatment team initiated dexamethasone 20 mg orally four times daily, along with anti-IL-1 and anti-IL-6 therapies to manage ICANS.

Her past surgical history included an enucleation a few years prior. She had no past psychiatric history. She denied tobacco, alcohol, or illicit drug use. Her family history was unremarkable.

During the psychiatric evaluation, the patient exhibited significant difficulty directing, sustaining, and shifting attention, requiring frequent prompting throughout the interview. She was oriented only to person and place. Memory difficulties were noted, particularly with delayed recall. Her thought process was tangential, reflecting a disorganized thought process. Due to these cognitive impairments and her limited ability to engage meaningfully during the interview, a formal cognitive screening could not be performed. No symptoms of delusions or hallucinations were elicited or observed.

Pertinent laboratory findings included neutropenia, anemia, and thrombocytopenia. The EEG findings were consistent with cortical hyperexcitability in the left parietal region with an increased risk for seizures and mild encephalopathy. No seizures were observed throughout the recording. Brain MRI showed a stable metastatic lesion in the right clivus and small enhancing areas in the right planum sphenoidal and inferior orbital gyrus/dura that were new/increased in size compared to prior examination, raising concern for brain metastasis.

The psychiatric assessment was consistent with hypoactive delirium, attributed to multiple medical etiologies. Delirium precautions were maintained, and gradual improvement was noted as dexamethasone was tapered from 20 mg four times daily to 4 mg oral daily, alongside ongoing anti-IL-1 and anti-IL-6 therapy. Collaborative care from the psychiatric and oncology teams played a key role in her recovery.

## 4. Discussion

This literature review and case series emphasize the clinical and pathophysiological complexities of ICANS, particularly its often-overlooked psychiatric manifestations. ICANS remains a significant challenge for individuals undergoing (CAR) T-cell therapy due to its variable presentation, ranging from subtle cognitive changes to severe neuropsychiatric symptoms.

CAR T-cell therapy, well known for its success in treating hematologic malignancies, is now being explored as a promising approach for solid tumors such as melanoma and NSCLC, particularly in patients who have developed resistance to standard therapies, including immune checkpoint inhibitors (ICIs) [7,8]. Despite its potential, the application of CAR T-cell therapy in solid tumors presents significant challenges, including tumor antigen heterogeneity, limited T-cell infiltration, and an immunosuppressive tumor microenvironment [7,8].

In melanoma, several antigenic targets, including c-MET, CD70, VEGFR2, and GD2, have been investigated due to their roles in tumor growth, angiogenesis, and immune evasion [9]. Resistance to ICIs remains a critical barrier to effective treatment, highlighting the need for novel therapeutic strategies such as CAR T-cell therapy. Similarly, in NSCLC, CAR T-cell therapy is being developed to target tumor-specific antigens, such as GPC3, PD-L1, and CD80/CD86, aiming to bypass resistance mechanisms and improve patient outcomes [10].

These advancements are particularly relevant to the cases presented, in which patients with melanoma and NSCLC experienced disease progression despite prior immunotherapy, including ICIs. Given the exhaustion of standard treatment options, CAR T-cell therapy was selected as a salvage treatment strategy in both cases, offering a novel approach to address their treatment-resistant disease.

The clinical features of ICANS typically appear between 3 and 10 days following the administration of CAR T-cell therapy, with symptoms often emerging 2 to 4 days after the onset of cytokine release syndrome (CRS), a common precursor to ICANS [1]. While most patients develop symptoms within this window, approximately 10% experience a delayed onset, presenting as late as three weeks after therapy initiation. ICANS encompasses a broad range of neurological and behavioral symptoms, including confusion, speech and language disturbances, headaches, attention deficits, and behavioral changes. Severe cases may present with seizures, cerebral edema, elevated intracranial pressure, or transient coma [1].

In a study by Mullane et al., long-term neuropsychiatric outcomes were evaluated in 40 survivors of CD19 CAR T-cell therapy [3]. Nearly half of the patients reported clinically significant symptoms, such as anxiety, depression, and cognitive difficulties, many of which were directly associated with ICANS. Among the cognitive impairments identified, patients exhibited difficulties in multiple domains, including memory deficits, impaired concentration, and challenges in solving complex problems. These findings highlight the potential for ICANS to disrupt multiple neurocognitive domains and adversely affect daily functioning and quality of life [3].

Maillet et al. described acute neurotoxicities following CAR T-cell therapy for relapsed/refractory B-cell lymphoma [4]. Patients with grade 2–4 neurotoxicity exhibited distinct cognitive impairments, including dyscalculia, dysgraphia, and executive dysfunction, reflecting the breadth of neurocognitive impact in more severe ICANS presentations [4]. Quinn et al. assessed neurotoxicity in 34 patients with B-cell lymphoma treated with axicabtagene ciloleucel [5]. Neurotoxicity occurred in 16 patients (47%), with 75% of cases resolving by discharge. However, 25% experienced prolonged effects, including confusion, disorientation, and persistent cognitive impairment lasting beyond one month. Two patients exhibited significant functional decline, and one transitioned to hospice care [5].

These findings emphasize the complex nature of CAR T-cell-associated neurotoxicity, which is marked by various cognitive impairments, including word-finding difficulty, executive dysfunction, and language disturbances, even in transient cases. The persistence of neurotoxicity in some patients underscores the importance of early detection, timely intervention, and targeted cognitive rehabilitation to improve long-term outcomes and quality of life. However, distinguishing ICANS-related neurocognitive deficits from those caused by underlying malignancies or concurrent treatment effects remains a significant clinical challenge.

The identification of biomarkers for ICANS is increasingly recognized as a crucial advancement for early detection, risk stratification, and treatment monitoring in patients undergoing CAR T-cell therapy. Biomarkers provide objective insights into the severity of neurotoxicity and help distinguish ICANS from other neurologic or psychiatric complications, enabling timely and targeted interventions.

One of the most promising biomarkers is neurofilament light chain (NfL), a structural protein released into circulation following axonal damage [11]. Elevated NfL levels in both serum and cerebrospinal fluid (CSF) have been strongly associated with more severe ICANS and may serve as a predictor of long-term neurocognitive impairment even after acute symptoms resolve [11]. As NfL can be measured non-invasively through serum sampling, it presents a valuable tool for monitoring neuronal injury and tracking patients for delayed cognitive dysfunction.

Recent research has explored the role of CSF biomarkers, which may provide a more direct assessment of central nervous system inflammation in ICANS. Elevated CSF protein levels, increased leukocyte counts, and elevated concentrations of inflammatory cytokines have been reported in ICANS patients, suggesting that CSF analysis could serve as a valuable diagnostic tool, particularly in cases where the clinical presentation overlaps with other neurologic conditions [12].

Advancements in neuroimaging and machine-learning models have further expanded the potential for biomarker-driven ICANS prediction. Functional MRI and diffusion tensor imaging have demonstrated structural and functional changes in ICANS patients, while EEG abnormalities, such as increased delta wave activity and epileptiform discharges, have been correlated with ICANS severity [13]. Emerging machine-learning approaches aim to integrate clinical, laboratory, and imaging data to develop predictive models that can identify high-risk patients and optimize treatment strategies.

While these biomarkers represent significant progress in understanding ICANS, further validation through large-scale studies is necessary to establish their clinical utility. The integration of biomarker-based approaches into standard ICANS management protocols could improve early intervention, reduce long-term neurological sequelae, and ultimately enhance the safety and efficacy of CAR T-cell therapy.

The exact mechanisms underlying ICANS remain unclear, though evidence suggests that inflammation induced by CAR T-cell therapy triggers endothelial activation, compromising the integrity of the blood–brain barrier and contributing to neurotoxicity [14,15]. Multiple risk factors have been associated with the onset of ICANS, including pre-existing neurological conditions, a prior occurrence of CRS, elevated CAR T-cell doses and peak expansion rates, high tumor burden at the time of infusion, reduced platelet counts, and elevated levels of inflammatory markers, such as C-reactive protein (CRP), ferritin, and cytokines including IL-1, IL-6, IL-10, and interferon-gamma [14,15].

A key event in ICANS pathophysiology is endothelial activation, which compromises the integrity of the BBB, enabling the influx of inflammatory cytokines and immune cells into the CNS. Biomarkers such as angiopoietin-2 (ANG-2) and von Willebrand factor (vWF), which are elevated during endothelial injury, have been linked to BBB disruption [16]. Increased levels of these markers suggest endothelial dysfunction and could be used as a predictor of severe ICANS. Identifying patients with elevated endothelial biomarkers could facilitate early risk stratification, enabling clinicians to implement preventive interventions before the onset of severe neurotoxicity [16]. Additionally, lactate dehydrogenase (LDH), commonly elevated in patients with high tumor burden, has been identified as an indirect predictor of ICANS. Elevated LDH levels prior to CAR T-cell infusion, reflecting high metabolic activity from tumor lysis or cellular stress, have been independently correlated with an increased risk of developing ICANS [17]. Beyond these established markers, research has highlighted novel associations between fibrinogen levels and ICANS risk. Specifically, elevated fibrinogen levels before infusion, followed by a significant decline from baseline to nadir during therapy, have emerged as important predictors of ICANS [17].

Cytokine profiling also provided valuable insights into the inflammatory mechanisms driving ICANS. Elevated levels of pro- and anti-inflammatory cytokines, including IL-6, IL-1β, IL-10, IFN-γ, and granulocyte-macrophage colony-stimulating factor (GM-CSF), have been detected in patients with ICANS, particularly in severe cases [18]. Among these, IL-6 and IL-1β, key mediators of systemic inflammation, are strongly implicated in BBB disruption, while GM-CSF, which promotes T-cell activation, has been directly linked to neuroinflammation [18]. Measuring these cytokines may serve as an early warning system for impending neurotoxicity, allowing clinicians to proactively adjust treatment strategies, such as initiating cytokine inhibitors to prevent progression to severe ICANS.

Beyond ICANS, those undergoing CAR-T therapy may experience a range of psychiatric manifestations, including anxiety, depression, and other mood disturbances [3]. These symptoms are thought to result from a combination of factors, such as the systemic inflammatory response triggered by CRS, the psychological burden of severe illness, and the stress associated with intensive treatment regimens. The pro-inflammatory cytokines involved in CRS, such as IL-6 and TNF-α, have been well implicated in the pathophysiology of mood and anxiety disorders, as inflammation is increasingly recognized as a contributor to psychiatric conditions [19].

The effective management of ICANS requires timely recognition, accurate grading, and a comprehensive treatment approach. In response to the growing need for standardization, the American Society for Transplantation and Cellular Therapy (ASTCT) introduced a grading system in 2019 to guide clinical management [20]. This grading system evaluates key neurological symptoms, including the level of consciousness, aphasia, motor impairments, seizures, and signs of elevated intracranial pressure, categorizing ICANS severity from mild to life-threatening [20]. Proper grading not only aids in risk stratification but also informs treatment strategies tailored to the severity of symptoms.

Management strategies for ICANS are guided by severity. Mild cases typically require supportive care, including frequent neurological assessments and symptomatic management. Moderate cases often benefit from corticosteroids, particularly dexamethasone, to reduce neuroinflammation. Severe cases, characterized by seizures or cerebral edema, necessitate aggressive treatment with high-dose corticosteroids, intensive care support, and cytokine inhibitors such as IL-1 receptor antagonists or siltuximab, an anti-IL-6 agent, particularly when cytokine release syndrome (CRS) is present [21,22]. Given the ongoing debate regarding the efficacy of IL-6 versus IL-1 inhibition in ICANS management, further research is needed to determine the most effective cytokine-targeting strategy. Early detection and prompt intervention are critical in reducing the risk of long-term neurological complications.

Corticosteroids remain the cornerstone of ICANS management. Dexamethasone and methylprednisolone are the most commonly used agents due to their ability to rapidly reduce neuroinflammation and restore blood–brain barrier integrity. Corticosteroids are particularly beneficial in cases of cerebral edema, elevated intracranial pressure, or seizures. Additionally, their ability to mitigate systemic inflammation makes them effective in managing concurrent CRS, which often precedes ICANS.

However, despite these benefits, corticosteroids are associated with significant psychiatric side effects, particularly at high doses or with prolonged use. Patients may experience mood disturbances, including anxiety, irritability, and severe depression. In some cases, steroid-induced psychosis, characterized by paranoia, hallucinations, and agitation, can develop. Individuals with pre-existing psychiatric disorders, such as mood disorders or schizophrenia, are at heightened risk of these complications. Additionally, cognitive impairments, including attention deficits and memory disturbances, may persist even after steroid tapering. The long-term use of corticosteroids also increases the risk of steroid-induced delirium, especially in elderly or hospitalized patients, contributing to prolonged hospital stays and reduced quality of life [23]. The careful titration of corticosteroids, close psychiatric monitoring, and non-pharmacological interventions such as structured routines and early mobilization are essential for minimizing these adverse effects. To reduce these risks, dexamethasone dosing should be carefully titrated, ensuring the lowest effective dose is administered for the shortest possible duration. This should be accompanied by regular mental status monitoring to detect changes in cognition or psychiatric symptoms.

Cytokine inhibitors offer an alternative or adjunctive treatment or to corticosteroids in managing ICANS, especially in severe or corticosteroid-refractory cases. IL-6 antagonists such as tocilizumab and siltuximab, and IL-1 inhibitors like anakinra, target inflammatory pathways implicated in ICANS pathophysiology [14,24]. IL-6 blockade has demonstrated rapid efficacy in resolving CRS, which often overlaps with ICANS, making it a preferred option in many cases [14,24]. Despite their effectiveness, cytokine inhibitors also carry neuropsychiatric risks. IL-6, a key cytokine in neurogenesis and synaptic plasticity, plays a vital role in normal brain function, and its inhibition has been associated with increased susceptibility to depression and cognitive impairments, including memory deficits and executive dysfunction [25]. The excessive suppression of immune responses from cytokine blockade also increases the risk of opportunistic infections, which can exacerbate delirium and other neuropsychiatric complications. Therefore, when using cytokine inhibitors, clinicians must balance their benefits with potential side effects, ensuring that patients are closely monitored for signs of cognitive decline.

Managing ICANS requires a multidisciplinary approach, integrating pharmacological interventions with supportive and rehabilitative care. Close collaboration among oncologists, neurologists, psychiatrists, and intensive care specialists ensures comprehensive monitoring, timely intervention, and the appropriate management of complications. Additionally, non-pharmacological strategies play an essential role, especially in preventing long-term neurocognitive and psychiatric complications.

Cognitive rehabilitation is a critical component of recovery for patients with persistent neurocognitive deficits following ICANS. Rehabilitation programs focus on enhancing memory, attention, and executive function through structured exercises and compensatory techniques [26]. Such interventions are particularly beneficial for patients experiencing prolonged difficulties with problem-solving, word retrieval, and processing speed, which can persist even after the acute phase of ICANS has resolved.

Environmental modifications are crucial for preventing and managing ICANS-related delirium. Strategies such as structured routines, optimized sleep hygiene, and minimizing sensory overstimulation help stabilize patients at risk of cognitive fluctuations. Sleep disturbances are common in critically ill patients and can exacerbate delirium and psychiatric symptoms. Interventions like sleep promotion, frequent reorientation, and early mobilization in hospital settings may reduce ICANS-related delirium.

Psychosocial support and multidisciplinary care are vital for comprehensive ICANS management. Given its complex neuropsychiatric effects, collaboration among oncologists, neurologists, psychiatrists, and rehabilitation specialists ensures holistic care. Educating families and caregivers facilitates the early detection of cognitive and behavioral changes while providing essential long-term support. Integrating psychological counseling, supportive therapy, and structured rehabilitation can help mitigate the emotional and cognitive burden associated with CAR T-cell therapy.

Combining these non-pharmacological strategies with pharmacological treatments enhances ICANS management, reducing long-term neuropsychiatric complications and improving patient outcomes.

The high prevalence of delirium during CAR T-cell therapy, particularly in the context of ICANS, underscores the need for early detection and proactive management. Both the hypoactive and hyperactive forms of delirium can significantly impact clinical outcomes, leading to prolonged hospitalization, cognitive decline, increased morbidity and mortality, and reduced quality of life. Potentially deliriogenic agents such as benzodiazepines, anticholinergic medications, and steroids should be carefully monitored. Implementing a structured approach, such as the ABCDEF bundle, can further enhance delirium prevention and management. This evidence-based framework incorporates regular pain assessment, minimizing unnecessary sedation, promoting early mobility, and involving family members in patient care. A multidimensional approach is essential to minimize delirium risk, enhance recovery, and optimize long-term cognitive and neurological outcomes while undergoing CAR T-cell therapy.

By incorporating these approaches, ICANS management can be further optimized to reduce the risk of long-term neuropsychiatric complications and improve overall patient outcomes.

The two case studies demonstrated varied psychiatric manifestations of ICANS, with both patients exhibiting delirium of differing presentations. The first case was characterized by hyperactive delirium with pronounced agitation and irritability, while the second case presented with hypoactive delirium. These cases highlight the complex and multifactorial pathophysiology of ICANS, which is influenced by cytokine release syndrome (CRS), blood–brain barrier dysfunction, and immune-mediated inflammation.

In Case Study 1, the patient’s clinical deterioration, despite seizure prophylaxis with levetiracetam and high-dose dexamethasone, suggests a significant inflammatory response contributing to the neuropsychiatric manifestations. Elevated lactate levels and thrombocytopenia further indicate systemic inflammation, which may have exacerbated neurotoxic symptoms.

In Case Study 2, the administration of anti-IL-1 and anti-IL-6 therapy alongside dexamethasone highlights the contribution of a systemic inflammatory response driven by excessive cytokine release, which leads to immune system overactivation and potential tissue damage in the progression of ICANS. This inflammatory cascade appears to contribute to cortical hyperexcitability, as evidenced by MRI findings suggesting a complex interplay between neuroinflammation and metastatic disease. Cortical hyperexcitability, characterized by increased neuronal activity in the cerebral cortex, heightens susceptibility to seizures, neuroinflammation, and other neurological complications. However, the presence of small enhancing areas in the right planum sphenoidale and right inferior orbital gyrus/dura raises concerns about progressive metastatic disease. Without CSF analysis, leptomeningeal involvement cannot be ruled out. Additionally, the patient’s pancytopenia may result from malignancy-related bone marrow suppression, treatment toxicity, or an underlying paraneoplastic process.

The EEG findings of left cortical hyperexcitability in the parietal region suggest an increased risk for seizure activity but do not definitively point to a single etiology. Inflammation from ICANS, steroid effects, direct metastatic infiltration, or a paraneoplastic encephalopathy could all contribute to the observed neuropsychiatric symptoms. The absence of anti-neuronal antibody testing is a limitation, as paraneoplastic neurological syndromes (PNSs) are often immune-mediated and may present with neuropsychiatric symptoms that mimic ICANS. Given these findings, a CSF analysis would have been valuable to test for malignant cells, inflammatory markers, and evidence of paraneoplastic autoimmunity.

The potential for multifactorial neurotoxicity, involving metastasis, systemic inflammation, and paraneoplastic mechanisms, must be carefully evaluated in the differential diagnosis. Given the presence of multiple coexisting medical conditions and treatment-related factors, attributing all neuropsychiatric symptoms solely to ICANS is challenging. Systemic inflammation, metastatic disease, corticosteroid effects, and metabolic or paraneoplastic complications represent significant confounders. Therefore, while ICANS remains a plausible contributor, a thorough assessment is essential to distinguish its impact from overlapping medical conditions, underscoring the importance of a multidisciplinary approach to diagnosis and management.

These two case studies underscore the diverse and complex neuropsychiatric manifestations of ICANS, highlighting the importance of comprehensive evaluation and individualized management. The diagnostic challenges presented by these cases emphasize the need for multimodal assessment, including advanced neuroimaging, biomarker analysis, and cerebrospinal fluid (CSF) evaluation, to differentiate ICANS from other overlapping conditions such as metastatic disease, paraneoplastic syndromes, and treatment-related toxicities. Additionally, the varying responses to anti-inflammatory and anti-cytokine therapies highlight the importance of individualized treatment strategies in managing ICANS. These cases also emphasize the critical role of a multidisciplinary approach, involving collaboration between oncology, neurology, psychiatry, and critical care teams, to ensure accurate diagnosis, mitigate complications, and optimize patient outcomes. Moving forward, further research and systematic biomarker profiling are essential to enhance the early detection, risk stratification, and treatment of ICANS, ultimately improving the safety and efficacy of CAR T-cell therapy in patients with advanced malignancies.

## 5. Conclusions

This study highlights how an improved recognition and comprehensive understanding of the manifestations associated with ICANS are essential to optimizing patient outcomes and mitigating the morbidity linked to this complex syndrome. Future research should prioritize longitudinal investigations to elucidate the progression and trajectory of neuropsychiatric symptoms in ICANS, as well as their potential long-term impacts on neurocognitive functioning and recovery. Furthermore, additional research is required to identify specific risk factors that predispose individuals to psychiatric complications related to ICANS and to develop targeted preventative and therapeutic strategies to reduce the incidence and severity of neuropsychiatric symptoms, ultimately enhancing the safety and effectiveness of CAR T-cell therapy.
